# Metallomic Profiling and Linkage Map Analysis of Early Parkinson's Disease: A New Insight to Aluminum Marker for the Possible Diagnosis

**DOI:** 10.1371/journal.pone.0011252

**Published:** 2010-06-22

**Authors:** Shiek S. S. J. Ahmed, Winkins Santosh

**Affiliations:** Department of Biotechnology, School of Bioengineering, SRM University, Kattankulathur, Tamil Nadu, India; Aristotle University of Thessaloniki, Greece

## Abstract

**Background:**

Parkinson's disease (PD) is the most common neurodegenerative disorder. The diagnosis of PD is challenging and currently none of the biochemical tests have proven to help in diagnosis. Serum metallomic analysis may suggest the possibility of diagnosis of PD.

**Methodology/Results:**

The metallomic analysis was targeted on 31 elements obtained from 42 healthy controls and 45 drug naive PD patients using ICP-AES and ICP-MS to determine the concentration variations of elements between PD and normal. The targeted metallomic analysis showed the significant variations in 19 elements of patients compared to healthy control (p<0.04). The partial least squares discriminant analysis (PLS-DA) showed aluminium, copper, iron, manganese and zinc are the key elements, contributes the separation of PD patients from control samples. The correlation coefficient analysis and element-element ratio confirm the imbalance of inter-elements relationship in PD patients' serum. Furthermore, elements linkage map analysis showed aluminium is a key element involved in triggering of phosphorus, which subsequently lead to imbalance of homeostatic in PD serum. The execution of neural network using elements concentrations provides 95% accuracy in detection of disease.

**Conclusions/Significance:**

These results suggest that there is a disturbance in the elements homeostasis and inter-elements relationship in PD patients' serum. The analysis of serum elements helps in linking the underlying cellular processes such as oxidative stress, neuronal dysfunction and apoptosis, which are the dominating factors in PD. Also, these results increase the prospect of detection of early PD from serum through neural network algorithm.

## Introduction

Parkinson's disease (PD) is a chronic neurodegenerative disorder, characterized by a progressive loss of substantia nigra pars compacta (SNc) neurons of unknown etiology [1]. The diagnosis of PD is entirely clinical with no biochemical tests presently available to diagnose PD. Current diagnosis is made by standard neurological examination and medical history. The severity of disease is categorized as stages based on the overall motor function evaluation using the Unified Parkinson's Disease Rating Scale (UPDRS) or Hoehn and Yahr scale or Schwab and England Activities of Daily Living Scale [2]. Three major cardinal symptoms of PD are tremor, rigidity and motor dysfunction, which significantly help in the detection of disease. However, the clinical diagnosis fails to identify the PD before the significant loss of dopamine neurons [3]. Hence, there is a need for early detection and more effective drugs to stop the progression of nigral degeneration [4].

Many decisions in medical practice are made by assessment of biomarkers. Biomarker serves as a diagnostic tool usually performed on body fluids, such as saliva, urine, serum or cerebrospinal fluid. Several studies suggest the diagnosis of PD from serum/plasma though the fundamental cause of disease is brain neurons [2], [5]. Metallomics approach is greatly focused on the detection of element markers for the diagnosis of diseases. Elements play a vital role in the biological system through their interaction with biomolecules. Elements regulate a number of cellular metabolic reactions, while a few of them act as etiological agents in many environmentally induced neurological disorders [6], [7]. Optimal concentration of elements is required for proper functioning of the human system. The deficiency of which causes serious metabolic abnormalities and increase leads to toxicity. The variations in serum elements can be potentially used for the diagnosis of diseases [8]. Several encouraging results were obtained using element analysis in an attempt to diagnose lung disease [9], hemochromatosis [10], chronic kidney disease [11], renal failure [12], cardiovascular disease [13] and liver disease [14].

In this study, an extensive analysis of 31 elements was made using inductive coupled plasma atomic emission spectroscopy (ICP-AES) and inductive coupled plasma mass spectroscopy (ICP-MS). The analysis was carried out on 42 normal and 45 drug-naive PD serum samples, to investigate the presence of variations in element concentrations of aluminium (Al), arsenic(As), barium (Ba), cadmium (Cd), calcium (Ca), cesium (Cs), chromium (Cr), cobalt (Co), copper (Cu), fluorine (F), iodine (I), iron (Fe), lead (Pb), mercury (Hg), magnesium (Mg), manganese (Mn), molybdenum (Mo), nickel (Ni), phosphorus (P), potassium (K), rubidium (Rb), selenium (Se), silicon (Si), silver (Ag), sodium (Na), strontium (Sr), sulphur (S), titanium (Ti), tungsten (W), vanadium (V) and zinc (Zn). The analysis was carried out on the early two stages of PD in order to determine the early diagnosis of disease. Also, the aim was to implement the concentrations of variable elements in artificial neural network (ANN) for early and rapid detection of PD. The results contribute to understand of metallomics dissimilarity patterns in PD compared to healthy control.

## Results

Investigation of serum samples for the detection of element variations between normal and PD patients were performed using ICP-AES and ICP-MS. The analysis of spectra aimed for 31 trace and ultra trace elements (Ag, Al, As, Ba, Ca, Cd, Co, Cr, Cs, Cu, F, Fe, Hg, I, K, Mg, Mn, Mo, Na, Ni, P, Pb, Rb, S, Se, Si, Sr, Ti, V, W and Zn).The elements were selected based on the previous elements interaction study [15], in order to make the element linkage map for PD. The trace and ultra trace elemental concentrations were expressed in terms of microgram/deciliter (µg/dL). The comparative analysis revealed the variations in 22 elements. Silver (Ag), cadmium (Cd), cobalt (Co), iron (Fe), rubidium (Rb), sulphur (S), selenium (Se) and zinc (Zn) were decreased and increased aluminium (Al), calcium (Ca), chromium (Cr), copper (Cu), mercury (Hg), potassium (K), magnesium (Mg), manganese (Mn), molybdenum (Mo), sodium (Na), nickel (Ni), phosphorus (P), lead (Pb) and vanadium (V) was noticed in PD (Table 1). The concentrations of these elements were ranged from 0.0071 µg/dL for cadmium to 321000 µg/dL for sodium in PD samples. The observed values obtained in this study was expressed as the mean of 42 normal and 45 PD samples together with standard deviation (SD) (Table 1).

**Table 1:** Significant variations in element concentration.

Elements	Concentrations (µg/dL)		
	Reference Value	Normal	Disease
Al	--	0.190±0.008	0.32±0.07
Ag	0.018	0.017±0.003	0.010±0.004*
Ca ^#^	9250	8450±720	10650±340
Cd	--	0.0119±0.0004	0.0071±0.006
Co	--	0.02±0.06	0.014±0.02
Cr	--	0.019±0.002	0.028±0.003*
Cu	75	83±8	98±3
Fe	119	123±8	110.4±0.6
Hg	--	0.136±0.021	0.199±0.03
K ^#^	15000	14200± 800	16300±300
Mg ^#^	1750	1750±20	2009±43
Mn	--	0.061± 0.01	0.076±0.004
Mo	0.15	0.09±0.05	0.19±0.02
Na^#^	312000	309000±3400	321000±1100
Ni	--	0.034±0.005	0.044±0.003
P ^#^	11900	10800±1200	14700±1600
Pb	--	0.048±0.003	0.069±0.002
Rb	17	15±3	01.7±0.7*
S	--	0.116±0.003	0.104±0.002
Se	17	17±3	1.8±0.4
V	--	0.005±0.0001	0.009±0.0002
Zn	65	59±7	43±4

*In significance (p>0.05); # trace analysis carried out using ICP-AES; -- Data not available.

### Statistical analysis

Analysis of variance (ANOVA) was performed to demonstrate the significance between the elements of control and PD (Table 1). In PD, Al, Cu, K, Mn, Mo, Na, P and V were increased significantly at p≤0.01 and Ca, Hg, Mg, Ni and Pb were increased significantly at p<0.05. The concentrations of Co, Fe, S, Se and Zn were decreased significantly at p≤0.01 and Cd was decreased significantly at p<0.05 in patient samples. The elements, Cr, Ag and Rb showed variations in concentrations with normal, were not attained the minimal significance of p≤0.05. The overall probability values of these 19 significant elements were ranged below 0.04 in PD serum compared to the control, which indicates the possible imbalance in elemental homeostasis.

### Element classification analysis

**Figure 1:**
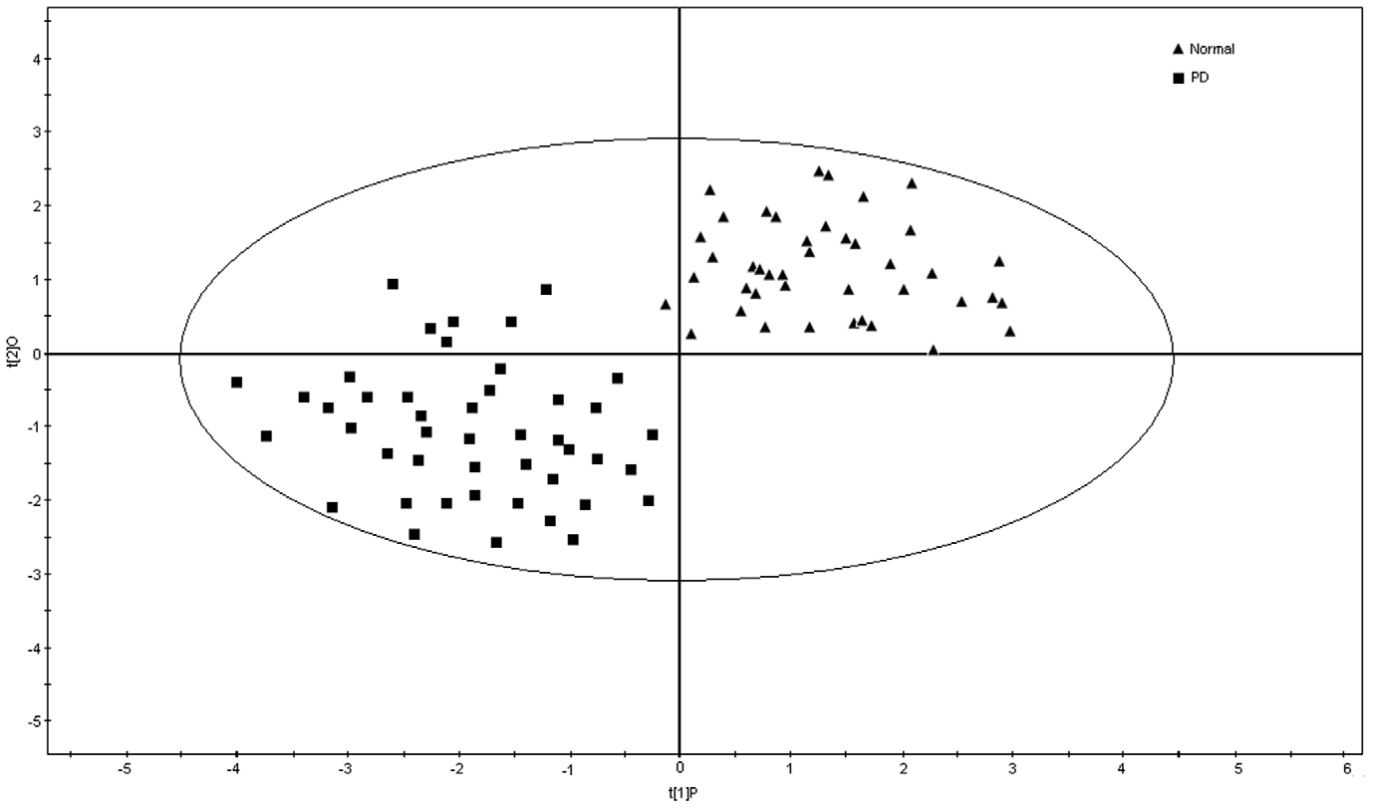
PLS-DA analysis of normal and PD serum elements. PLS-DA score's plot displays a significant separation between control subjects (n = 42) and unmedicated PD patients (n = 45) using complete digital maps. The observations coded according to class membership: triangle is normal and rhombus is PD. Each data point on a plot represents one individual.

**Figure 2:**
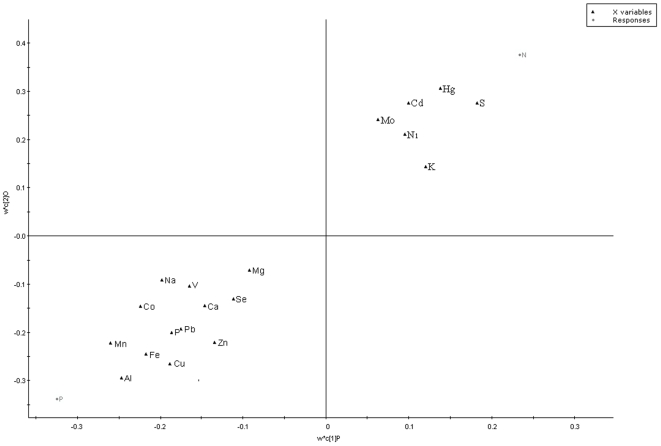
Biomarker detection: Elements which are dominating the separation of disease from normal. The loading coefficient map showing that aluminum, copper, iron, manganese, and zinc were predominantly responsible for the classification of groups. The blackened triangle represents elements; grey circle represents normal (N) and Parkinson's disease (P).

To explore the elements multidimensional data, unsupervised PLS-DA method was executed. The PLS-DA analysis was carried out on 19 significant variable elements of 87 samples. The analysis showed a clear differentiation between healthy volunteers and drug-naive patients (Fig. 1). The loading coefficient map indicates Al, Cu, Fe, Mn and Zn were dominantly responsible for the separation of PD from normal (Fig. 2). Hence, the PLS-DA of elements confirms the likely importance of elevated Al, Cu and Mn and decreased concentrations of Fe and Zn for the diagnosis of PD.

### Element inter- relationship

In order to bring out the inter-relationship between the major contributing elements, the element-element concentrations ratios was executed. The element-element ratios help to understand the interdependency of elements in the biological system. The results suggest that the ratios of Al/Cu, Al/Fe, Al/Mn, Al/Zn, Cu/Fe, Cu/Zn, Fe/Zn and Mn/Zn were increased and Fe/Mn and Cu/Mn were decreased in PD compared to healthy control (Table 2).

**Table 2:** Homeostatic imbalance of elements in comparison with control.

Elements X	Elements Y	Element ratio in normal	Element ratio in PD
Al	Cu	0.0022	0.0032
Al	Fe	0.0013	0.0028
Al	Mn	3.245	4.210
Al	Zn	0.0030	0.007
Cu	Fe	0.597	0.864
Cu	Mn	1459	1289
Cu	Zn	1.36	2.27
Fe	Mn	2442	1492
Fe	Zn	2.292	2.637
Mn	Zn	0.00093	0.0017

### Linkage map analysis

**Figure 3:**
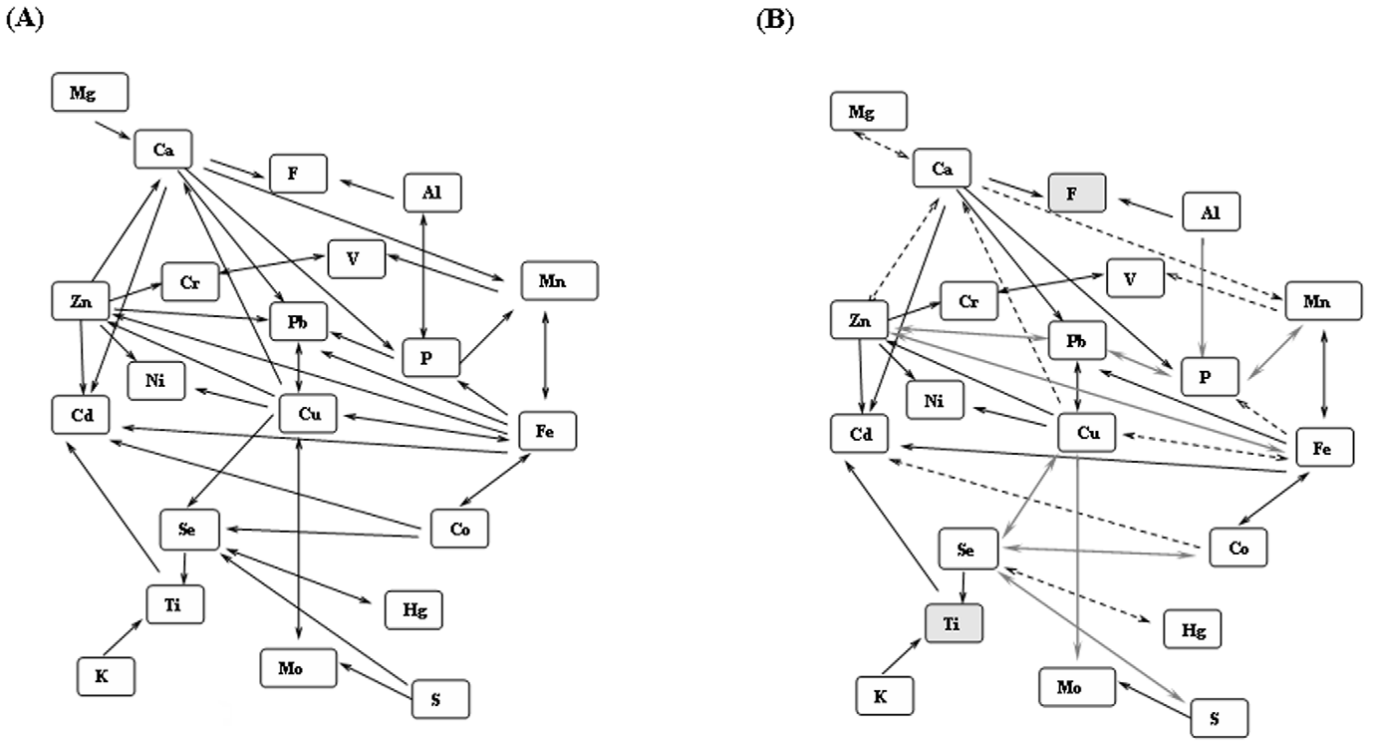
Element linkage map representing the interactions of elements. The interactions of 21 elements were configured. (A) Represents the basic interaction occurs in mammals. (B) Represents the interactions in PD. The single head arrow indicates the increase of an element X, decrease the element Y (negative correlation). The double headed arrow indicates, increase of an element X will increase the element Y or vice versa (positive correlation). The blackened arrow indicates similar significant patterns in (A) and (B); gray arrows indicate significant variations in interaction in PD and blackened dotted arrows indicate insignificant interaction. The grey box indicates insignificant variation in concentration between normal and disease (ANOVA), and their interactions analysis was not carried out.

The elements linkage map for PD was created using the correlation coefficient values of 18 significant elements with the understanding of previous elements interaction study [15]. Correlation coefficients were calculated using the concentrations (µg/dL) of 18 significant elements of PD. Implementation of PD correlation values in linkage map showed the variations in interaction pattern between Cu vs (Fe and Mo), P vs (Mn, Pb and Al), Se vs (Cu, Co and S), Zn vs (Pb, Fe and Ca), Ca vs Mg and Mn vs V in PD compared to previous elements interaction model (Fig. 3). In addition, few of the elements interactions are insignificant (p>0.05) in correlation analysis (Table 3).

**Table 3:** Element inter-relationship in PD for element linkage map.

Number of interaction	Element X	Element Y	Degree of correlation in PD
1	Ca	Mg	0.234*
2	Ca	P	−0.789
3	Cu	Mo	−0.397
4	Cu	Cd	−0.802
5	Cu	Ni	−0.973
6	Cu	Zn	0.922
7	Cu	Ca	−0.267*
8	Cu	Pb	0.737
9	Cu	Fe	0.289*
10	Cd	Co	−0.188*
11	Cd	Fe	−0.802
12	Cd	Ca	−0.359
13	Cd	Zn	−0.970
14	Fe	Mn	0.397
15	Fe	Pb	−0.433
16	Fe	P	−0.338*
17	Fe	Zn	0.636
18	Fe	Co	0.866
19	Mn	P	0.729
20	Mn	V	0.0821*
21	Mn	Ca	−0.155*
22	P	Al	−0.957
23	Pb	P	0.994
24	Pb	Ca	−0.848
25	S	Mo	−0.755
26	Se	Cd	−0.970
27	Se	Cu	0.636
28	Se	Co	0.419
29	Se	Hg	0.090*
30	Se	S	0.907
31	Zn	Ni	−0.986
32	Zn	Pb	0.419
33	Zn	Ca	0.124*

*Insignificance (p>0.05).

### ANN model and its diagnostic performance

**Figure 4:**
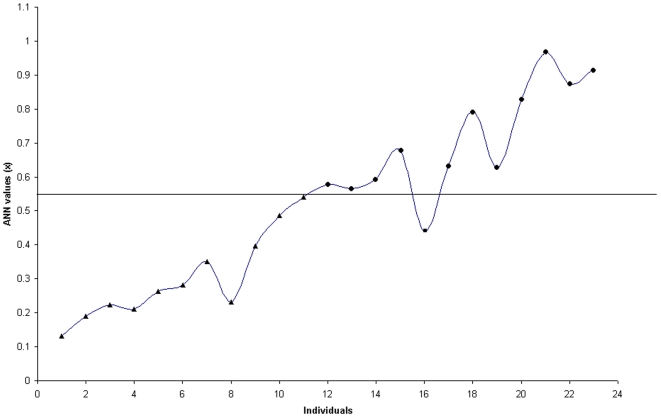
Neural network predication. Neural network classification of 23 individuals (x-axis) with known clinical information. Values (y-axis) are predicted over the trained network and are 0.1 to 1; values (x) ≤0.54 reflect a neural-network classification of “normal,” and values (x) ≥0.55 reflect a neural-network classification of PD. Individuals denoted by a triangle  =  normal, circle  =  PD and square  =  misdiagnosed individual.

The ANN model described in methodology was tested with 23 samples. The results were compared with known clinical status. The misclassification of one individual was noticed (Fig. 4). The success rate of the classification network was 95% (22/23) accuracy, whereas the specificity was 100% (11/11) and sensitivity was 91% (11/12). Furthermore, the values generated by the network are ranged from 0.1 to 1.0. Based on these values the individuals were assigned as normal or PD. The individuals whose final predicted values (x) ≤0.54 were assigned as normal and the values (x) ≥0.55 were assigned as PD.

## Discussion

Neurodegeneration in PD is a complex and multifaceted process, affects a specific population of nerve cells. Elements play a crucial role in the pathogenesis of neurodegeneration [16]–[22]. Several studies suggest that there is an elemental variation in serum/plasma of PD [23]–[26]. However, there is an argument in levels of variation in elements among these studies, which may be due to a population variation. On understanding of those variations, this study was focused on 42 normal and 45 PD affected patients of South Indian population. The analysis was carried out on 31 trace and ultra trace elements in the prospective of diagnosis through neural-network algorithm. Targeted metallomic analysis of serum showed differential distribution of 19 elements in drug naive patients compared to the healthy control and reference elemental values (Table 1) [27]. Also, PLS-DA analysis showed Al, Cu, Fe, Mn and Zn are the key elements significantly contribute for the separation between the metallomics profiles of unmedicated PD patients and controls (Fig. 2). Moreover, it was hypothesized that increased Al concentration affects concentration of other elements by increasing the concentration of Cu, Na, K, P and Mn and decreasing the concentration of Fe, S, and Zn in serum [28]. Similar variation patterns were also exhibited in this study

The serum Al level was significantly increased in PD patients. The Al concentration in our present investigation of control is 0.190 µg/dL and 0.32 µg/dL for PD. Al is a neurotoxin involved in Aβ aggregation, neuronal apoptosis and memory loss in animal model [29], [30]. The elevated serum Al concentration exhibits the clinical symptom of depression [28]. Increased Al was found to increase superoxide dismutase (SOD) activity to protect the cell from oxidative stress [31]. The increased SOD was previously reported in PD [32]. Furthermore, Al affects the integrity and functionality of mitochondria and endoplasmic reticulum (ER), which activates caspase-12 [33] that leads to endoplasmic mediated cell death [34]. Our results also demonstrate the increase Cu and decrease Zn. Similar trends were reported in schizophrenic, bipolar and unipolar patients [28], [35]–[38]. Cu is an essential element for the activity of cytochromeC oxidase, Cu/Zn superoxide dismutase and dopamine-beta-hydroxylase, which is critical in scavenging reactive oxygen species. The increased Cu generates reactive oxygen species which leads to oxidative stress and contributes to the cell death pathway [39]. Additionally, the serum Mn level was significantly increased in PD patients. Mn is associated with mitochondrial dysfunction and DNA fragmentation of primary striatal neurons [40]. The malfunctioning of mitochondria was reported [2] and DNA fragmentation mediated cell death was still unclear in PD.

Our results demonstrate the decrease concentrations of Fe with increase concentrations of Al in PD serum. Interestingly, similar results were obtained in patients suffering from chronic fatigue syndrome which affects cognition and influences neurological abnormalities. Also, Fe plays a key role in oxidative stress [41] and readily reduces hydrogen peroxide to liberate the reactive and unselective hydroxyl radical, capable of inflicting severe oxidative damage [42]–[44]. H_2_O_2_ is freely permeable across membranes and readily inflicts oxidative damage to cells. Fe mediated increased radicals promote mitochondrial dysfunction, oxidative stress and neuron dysfunction [45]. Supportive of this finding, the animal experiment showed an association between reduced Fe and dopaminergic neurons [46].

Hence metallomics analysis of this study results in the imbalance of elements concentrations leads to biochemical changes such as oxidative stress, mitochondrial dysfunction and neuronal death in PD. This elemental imbalance in serum may reflex in the elemental concentrations of Zn, Al and Pb in the brain which have been implicated in the aggregation of alpha-synuclein, a crucial protein in Parkinson's disease [47]. Also, it is notable that there is an inter relationships between the elements that maintain the homeostasis of biological system. Moreover, the abnormalities of a single element concentration will affect the total element distribution pattern in the biological system. For instance, the results of element-to-element ratio of major contributing elements showed the increase in ratios of Al/Cu, Al/Fe, Al/Mn and Al/Zn in patients compared to control (Table 2). The abnormality in elemental ratios may be due to increase in Al, which disturbs the element homeostasis in serum by increasing the paramagnetic oxidant elements like Cu and Mn while decreasing Zn, an antioxidant metal required as a cofactor for CuZn–SOD and Zn–thionein [28]. Furthermore, interdependency between the elements was validated using elements linkage map of previous study [15].

The elements linkage map of PD was created by implementing the significant correlation values of variable elements of PD in the linkage map model. Of 19 significant elements 18 were configured for linkage map analysis (Fig. 3). The element Na was excluded from the interaction study due to lack of interaction data in the model. The results show that the linkage map of previous model and PD patients were similar although there were variations in certain interaction pattern in PD patients. Nine significant variations in interaction patterns were noticed in PD (Fig. 3). Elements interaction between Cu vs Mo, P vs (Mn, Pb and Al), Se vs (Cu, Co and S) and Zn vs (Pb and Fe) were altered as shown in Figure 3. The interactions of F vs (Ca and Al) and Ti vs K were not measured due to the insignificant concentration of F and Ti in PD compared to healthy control.

In consideration of the Al hypothesis in PD linkage map (Fig. 3), Al was observed to have interaction with F and P. However, there is no significant variation in concentration of F was noticed in PD compared to normal. Hence, it is understandable that Al involved in triggering of P in PD. Subsequently, P interacts with the Fe and Mn and altered their concentrations significantly. Furthermore, the concentrations of Cu and Zn may be altered by P via Pb. Also, the concentration variations in these Cu and Zn altered Se and its interacting elements. Hence, Al mediated interaction of P plays a vital role in the homeostasis of PD and increased serum Al concentration might be related with the risk of PD.

The trace elements based diagnosis of PD was carried out using ANN. The results showed there is a misclassification of one diseased individual as normal (Fig. 4). The misclassification of this pathological condition may be due to the variable data of age and gender. The other variables of ANN were shown to be significant in ANOVA and PLS-DA. Hence, age and gender may be the major contributing factors for diagnosis. Few successful studies also showed the relation of gender and age with PD [48], [49]. However, the ANN diagnosis of PD provides 95% accuracy, which is more accurate than the current clinical diagnosis [2].

In conclusion, the present study of targeted metallomic profiling showed the significant abnormalities in 19 circulating elements, which help in linking the underlying cellular processes such as oxidative stress, neuronal dysfunction and apoptosis, the dominating factors in PD. Encouraging results were obtained in neural network for rapid detection of PD. Further work needs to be carried out in various populations to confirm the significance of the neural network in detection of PD. However, in present status it is suggested to execute a neural network along with a clinical diagnosis, which will additionally improve the accuracy of PD detection.

## Materials and Methods

### Sample collection

Clinical samples were collected from the out-patient setting of the Department of Neurology at SRM Hospital, Tamil Nadu, India. Drug-naive samples of 45 early PD patients were collected and 42 samples of age and gender-matched healthy controls were included for comparative study (Table 4). The written consent was obtained from each participant during in-person interview and blood donation. Data on gender, age, body mass index (BMI) and family history of PD were recorded. All individuals chosen for this study had no history of smoking, alcoholism, drug abuse and antioxidant treatment for a period of seven months before sample collection. The ethical committee of SRM Medical College Hospital & Research, India reviewed and approved the protocol of this study (Ref. No. 3496/Dean/07). The pathological status was well studied by a qualified neurologist and the disease stages were classified by UPDRS.

**Table 4:** Statistics of research participant's involved in study.

Sample information	Number of samples	Mean age in years±SD	Gender	
			Male	Female
Normal	42	55.62±3.25	25	17
*PD*	45	57.62±9.10	26	19

### Collection and storage of samples

Blood samples were collected using intravenous canula from individuals and immediately centrifuged for five min at 14,000 rpm to separate serum from other cellular materials. Serum separations were carried out under HEPA filtered-air condition and tubes used were polypropylened and no glass material was used to prevent Al and Si contaminations. All precautions to eliminate metal contamination during blood collection and storage were taken in accordance with the National Committee for Clinical Laboratory Standards (NCCLS) criteria [50]. Subsequently, serum was transferred to fresh tube for the ICP-AES and ICP-MS preparation. The serum was digested with 0.5 ml conc. HNO_3_ and dried using a hot air oven. The dried serum was further dissolved with 5 ml of 0.1 M HNO_3_ solution containing Ge, Rh and Re as internal standard. This solution was immediately subjected to ICP-AES and ICP-MS to avoid contaminations.

### ICP-AES and ICP-MS Instrumentation

The determination of Na, K, P, Ca and Mg in the serum samples was carried out using ICP-AES. The ultra trace elements were determined using ICP-MS instrument of Model SPQ8000A (Seiko Instruments, Chiba), equipped with quadruple mass spectrometer. The QA/QC of the instruments was performed to confirm and to validate test methodology including precision, accuracy and verifiable detection limits before the execution of analysis. Quality control was performed by analyzing a serum matrix matched multi-element synthetic standard and certified standard reference material obtained from the National Bureau of Standards, USA [51]. The instrumental and operating conditions are summarized in Table 5. The wavelengths of the emission line and mass numbers for the analyte elements were used in ICP-AES and ICP–MS for measurement, respectively. The lines were selected for each element in such a way that interference from the other elements was at minimum.

**Table 5:** The ICP instrumental and operating conditions.

Instrument parameter	Optimization
**ICP- AES**	
***Gas Conditions***	
Carrier gas	Ar 18 l/min
Outer gas	Ar 1.03 l/min
Intermediate gas	Ar 0.57 l/min
***Plasma conditions***	
Rf frequency	27.12 MHz
Incident Rf power	1 kW
***Sampling conditions***	
Observation height	18 mm above work coil
Sampling uptake rate	1.1 ml/min
***Torch***	Fassel type
***Spray chamber***	Single type
***Nebulizer***	Cross-flow type
**ICP- MS Instrument**	
***Gas Conditions***	
Carrier gas	Ar 16 l/min
Outer gas	Ar 1.04 l/min
Intermediate gas	Ar .95 l/min
***Plasma conditions***	
Rf frequency	27.12 MHz
Incident Rf power	1 Kw
***Sampling conditions***	
Sampling depth	12 mm from work coil
Sampling uptake rate	0.8 ml/min
Sampling cone	Copper, 1.1 mm orifice diameter
Skimmer cone	Copper, 0.35 mm orifice diameter
***Data acquisition***	
Accumulation	20 times
Dwell time	10 ms/point
Repetition	5 times
Channel width	3 channels
***Torch***	Fassel type
***Spray chamber***	Scott type
***Nebulizer***	Concentric type

### Statistical analyses

The elements profiled data was imported into GeneSpring GX7.3 microarray software (Agilent Technologies Inc., Santa Clara, California), in which the analysis of variance (ANOVA) was performed. In order to confirm the biomarkers differentiating the patients from matched controls, PLS-DA was employed using Umatrices software (Umetrics, Inc., Kinnelon, NJ). The correlation coefficient and element-to-element ratios were calculated using Microsoft Excel 2003. The PD network map was created using the correlation coefficient values and mammalian network of previous study [15].

### Artificial Neural network (ANN)

A NeuNet Pro software (CorMac Technologies Inc., Canada) was used for the ANN prediction. The ANN adopted in this study was three-layered network of back propagation algorithm. The input layer consisted of seven neurons (age, gender and concentrations of aluminium, copper, iron, manganese and zinc). The hidden layer, with six units, and an output layer, whose output values ranged from 0 to 1 indicating the likelihood of PD. The network was trained with variable data of randomly selected 64 individuals (31 normal and 33 PD) from the set of 87, with known pathological information. The training process continued until the difference between the ANN classification and clinical diagnosis became diminished. Once the network was trained, the remaining 23 individuals were tested using the trained network. The outcome of classification results was then compared to clinical data to determine the classification ability of the network.

## References

[1] Guttman M, Kish SJ, Furukawa Y (2003). Current concepts in the diagnosis and management of Parkinson's disease.. CMAJ.

[2] Ahmed SSSJ, Santosh W, Kumar S, Christlet HTT (2009). Metabolic profiling of Parkinson's disease: evidence of biomarker from gene expression analysis and rapid neural network detection.. J Biomed Sci.

[3] Ahmed SSSJ, Santosh W, Kumar S (2009). Neural network algorithm for the early detection of Parkinson's disease from blood plasma by FTIR micro-spectroscopy..

[4] Gibb WR, Lees AJ (1988). The relevance of the Lewy body to the pathogenesis of idiopathic Parkinson's disease.. J Neurol Neurosurg Psychiatry.

[5] Gelb, Oliver, Gilman (1999). Diagnostic criteria for Parkinson disease.. Arch Neurol.

[6] Strong MJ, Garruto RM, Woodruff ML, Nonneman AJ (1994). Experimental paradigms of motor neuron degeneration.. Toxin-induced Models of Neurological Disorders.

[7] Garruto RM, Flaten TP, Wakayama I, Corain B, Wisneiwski H, Zatta P (1993). Natural and experimental models of environmentally induced neurodegeneration: Implication for Alzheimer's disease.. Alzheimer's Disease: Advances in Clinical and Basic Research.

[8] Takuya H, Kazumi I, Hiroki H (2001). Multielement Correlation Analysis of Major-to-Trace Elements in Human Blood Serum for Medical Diagnosis as Studied by ICP-AES and ICP-MS.. Anal Sci.

[9] Koichi W, Masayoshi K (2003). Trace Elements in the Environment The Elements Analysis of the Tissue Section of the Lung Disease Caused by Environmental Pollution by Means of Electron Probe Microanalysis.. Biomed Res Trace Elem.

[10] Hagve TA, Asberg A, Ulvik R, Borch-Iohnsen B, Thorstensen K (2009). Hemochromatosis from an underdiagnosed curiosity to a common disease.. Tidsskr Nor Laegeforen.

[11] Nikolov IG, Mozar A, Drüeke TB, Massy ZA (2009). Impact of disturbances of calcium and phosphate metabolism on vascular calcification and clinical outcomes in patients with chronic kidney disease.. Blood Purif.

[12] Hur CI, Yoon TR, Cho SG, Song EK, Seon JK (2008). Serum ion level after metal-on-metal THA in patients with renal failure.. Clin Orthop Relat Res.

[13] Park W, Kim BS, Lee JE, Huh JK, Kim BJ (2009). Serum phosphate levels and the risk of cardiovascular disease and metabolic syndrome: a double-edged sword.. Diabetes Res Clin Pract.

[14] Harrison-Findik DD (2009). Is the iron regulatory hormone hepcidin a risk factor for alcoholic liver disease?. World J Gastroenterol.

[15] Seiler HG, Sigel A, Sigel H, Seiler G, Sigel A, Sigel H (1994). Overview And use of the hand book.. Hand book on metals in clinical and analytical chemistry.

[16] Cuajungco MP, Lees GJ (1997). Zinc and Alzheimer's disease: is there a direct link?. Brain Res Brain Res Rev.

[17] Basun H, Forssell LG, Wetterberg L, Winblad B (1991). Metals and trace elements in plasma and cerebrospinal fluid in normal aging and Alzheimer's disease.. J Neural Transm Park Dis Dement Sect.

[18] Zecca L, Pietra R, Goj C, Mecacci C, Radice D (1994). Iron and other metals in neuromelanin, substantia Nigra and Putamen of human brain.. J Neurochem.

[19] Rao KSJ, Rao RV, Shanmugavelu P, Menon RB (1999). Trace elements in Alzheimer's brain: A new hypothesis.. Alz Rep.

[20] Bharathi, Vasudevaraju, Govidaraju, Palanisamy, Sambamurti (2008). Molecular toxicity of aluminum in relation to neurodegeneration.. Indian J Med Res.

[21] Gaeta A, Hider RC (2005). The crucial role of metal ions in neurodegeneration: the basis for a promising therapeutic strategy.. Br J Pharmacol.

[22] Bharathi, Ravid R, Rao KS (2006). Role of Metals in Neuronal Apoptosis: Challenges Associated with Neurodegeneration.. Curr Alzheimer Res.

[23] Hegde ML, Shanmugavelu P, Vengamma B, Rao TS, Menon RB (2004). Serum trace element levels and the complexity of inter-element relations in patients with Parkinson's disease.. J Trace Elem Med Biol.

[24] Alimonti A, Ristori G, Giubilei F, Stazi MA, Pino A (2007). Serum chemical elements and oxidative status in Alzheimer's disease, Parkinson disease and multiple sclerosis.. Neurotoxicology.

[25] Abbot RA, Cox M, Markus H, Tomkins A (1992). Diet, body size and micronutrient status in Parkinson's disease.. Eur J Clin Nutr.

[26] Jimenez-Jimenez FJ, Fernandez-Calle P, Martinez-Vanaclocha M, Herrero E, Molina JA (1992). Serum levels of zinc and copper in patients with Parkinson's disease.. J Neurol Sci.

[27] Hasegawa T, Matsuura H, Inagaki K, Haraguchi H (2003). Major-to-ultratrace element in bone marrow fluid as determined and ICP-MS.. Anal Sci.

[28] Mustak MS, Rao TS, Shanmugavelu P, Sundar NM, Menon RB (2008). Assessment of serum macro and trace element homeostasis and the complexity of inter-element relations in bipolar mood disorders.. Clin Chim Acta.

[29] Bharathi, Shamasundar NM, Sathyanarayana Rao TS, Dhanunjaya Naidu M, Ravid R (2006). A new insight on Al-maltolate-treated aged rabbit as Alzheimer's animal model.. Brain Res Rev.

[30] Savory J, Rao JK, Huang Y, Letada PR, Herman MM (1999). Age-related hippocampal changes in Bcl-2:Bax ratio, oxidative stress, redox-active iron and apoptosis associated with aluminum-induced neurodegeneration: increased susceptibility with aging.. Neurotoxicology.

[31] Kuloglu M, Ustundag B, Atmaca M, Canatan H, Tezcan AE (2002). Lipid peroxidation and antioxidant enzyme concentrations in patients with Schizophrenia and bipolar disorder.. Cell Biochem Funct.

[32] Kalra J, Rajput AH, Mantha SV, Prasad K (1992). Serum antioxidant enzyme activity in Parkinson's disease.. Mol Cell Biochem.

[33] Savory J, Herman MM, Ghribi O (2003). Intracellular mechanisms underlying aluminium-induced apoptosis in rabbit brain.. J Inorg Biochem.

[34] Ghribi O, Herman MM, Dewitt DA, Forbes MS, Savory J (2001). A beta (1–42) and aluminium induce stress in the endoplasmic reticulum in rabbit hippocampus, involving nuclear translocation of gadd 153 and NF-kappa B.. Brain Res Mol Brain Res.

[35] Olatunbosun DA, Akindele BK, Adadevoh BK, Asuni T (1975). Serum copper in schizophrenia in Nigerians.. Br J Psychiatry.

[36] Alias AG, Vijayan N, Nair DS, Sukumaran M (1972). Serum ceruloplasmin in schizophrenia:significant increase in acute cases especially in catatonia.. Biol Psychiatry.

[37] Pfeiffer CC, Ilier UA (1972). Study of zinc deficiency and copper excess in the schizophrenias.. Int Rev Neurobiol.

[38] Diebel MA, Ehmann WD, Markesbery WR (1996). Copper, iron and zinc imbalances severely degenerated brain regions in Alzheimer's disease: possible relation to oxidative stress.. J Neurol Sci.

[39] Carri MT, Ferri A, Cozzolino M, Calabrese L, Rotilio G (2003). Neurodegeneration in amyotrophic lateral sclerosis: the role of oxidative stress and altered homeostasis of metals.. Brain Res Bull.

[40] Malecki EA (2001). Manganese toxicity is associated with mitochondrial dysfunction and DNA fragmentation in rat primary striatal neurons.. Brain Res Bull.

[41] Huang X, Cuajungco MP, Atwood CS, Hartshorn MA, Tyndall JD (1999). Cu (II) potentiation of alzheimer abeta neurotoxicity. Correlation with cell-free hydrogen peroxide production and metal reduction.. J Biol Chem.

[42] Bush AI, Pettingell WH, Multhaup G, deParadis M, Vonsattel JP (1994). Rapid induction of Alzheimer Abeta amyloid formation by zinc.. Science.

[43] Atwood CS, Moir RD, Huang X, Scarpa RC, Bacarra NM (1998). Dramatic aggregation of Alzheimer Ab by Cu(II) is induced by conditions representing physiological acidosis. J. Biol.. Chem.

[44] Bush AI, Multhaup G, Moir RD, Williamson TG, Small DH (1993). A novel zinc(II) binding site modulates the function of the beta A4 amyloid protein precursor of Alzheimer's disease.. J Biol Chem.

[45] Savory J, Rao JK, Huang Y, Letada PR, Herman MM (1999). Age-related hippocampal changes in Bcl-2: Bax ratio, oxidative stress, redox-active iron and apoptosis associated with aluminum-induced neurodegeneration: increased susceptibility with aging.. Neurotoxicology.

[46] Youdim MBH, Yehuda, Ben-Shackar D, Ashkenazi R, Pollitt E, Leibel RL (1982). Behavioral and brain biochemical changes in iron deficient rats: the involvement of iron in dopamine receptor function.. Iron deficiency: brain biochemistry and behavior.

[47] O'Keeffe GC, Michell AW, Barker RA (2009). Biomarkers in Huntington's and Parkinson's Disease.. Ann N Y Acad.

[48] Wooten GF, Currie LJ, Bovbjerg VE, Lee JK, Patrie J (2004). Are men at greater risk for Parkinson's disease than women?.. J Neurol Neurosurg Psychiatry.

[49] Haaxma CA, Bloem BR, Borm GF, Oyen WJ, Leenders KL (2007). Gender differences in Parkinson's disease.. J Neurol Neurosurg Psychiatry.

[50] National Committee for Clinical Laboratory Standards Approved Guidelines. Available: http://www.clsi.org/source/orders/free/c38-a.pdf via the Internet. Accessed 12 Feb 2008

[51] Gillian L, Jack DF, Benjamin G, David EN, Patrick JP (1997). Control of pre-analytical variation in trace element determination.. National committee for Clinical Laboratory Standards Approved guidelines.

